# HEALTH CARE UTILIZATION BEFORE AND AFTER ENROLLMENT IN HOME-BASED PRIMARY CARE

**DOI:** 10.1093/geroni/igae098.3345

**Published:** 2024-12-31

**Authors:** Samantha Rizzo, Anne Wahl, Thomas Chameli, Lindsay Ayers, Parsa Mahmoudi, Yijung Kim, Lauren Bangerter, Karl De Jonge

**Affiliations:** Beth Israel Deaconess Medical Center, Brookline, Massachusetts, United States; Washington Hospital Center, Washington, District of Columbia, United States; Georgetown University School of Medicine, Washington, District of Columbia, United States; Georgetown University School of Medicine, Washington, District of Columbia, United States; Georgetown University School of Medicine, Washington, District of Columbia, United States; Medstar Health Research Institute, Hyattsville, Maryland, United States; Medstar Health, Hyattsville, Maryland, United States; MedStar Washington Hospital Center, Washington, District of Columbia, United States

## Abstract

Homebound older adults typically exhibit higher rates of emergency department (ED) visits and hospital admissions compared to non-homebound individuals. This study evaluated health care utilization rates before and after patients enrolled in the MedStar Washington Hospital Center House Call Program, a home-based primary care (HBPC) model. Data were drawn from the electronic medical record for 12-months before and 12-months after enrollment in the HBPC program. A total of 530 patients enrolled in HBPC between June 30, 2018, and July 1, 2023. Most patients were female (76.4%) and African American (80.8%), with an average age of 84.3 at enrollment. Over 40% of the patients were living with dementia, 23% had experienced a stroke, and 81% had hypertension. Using paired t-tests, we found a decrease in the average annual rate of ED visits (1.58 vs. 0.92, p < 0.001) and hospital admissions (0.91 vs. 0.53, p < 0.001) from the pre-12-month enrollment period to the post-12-month period. Linear regression revealed important predictors of post-enrollment ED visits, including greater pre-enrollment ED use, history of chronic obstructive pulmonary lung disease (COPD), and coronary artery disease (CAD). Post-enrollment hospital admissions were predicted by the frequency of pre-enrollment admissions, presence of chronic kidney disease (CKD), and CAD. Patients with CAD, CKD, or COPD are more likely to present to the ED or hospital during enrollment in HBPC than those without these comorbidities The overall utilization results suggest that enrollment in a HBPC model lessens the rate of ED visits and hospital admissions in a homebound population.

